# Yedoma Permafrost Releases Organic Matter with Lesser Affinity for Cu^2+^ and Ni^2+^ as Compared to Peat from the Non-Permafrost Area: Risk of Rising Toxicity of Potentially Toxic Elements in the Arctic Ocean

**DOI:** 10.3390/toxics11060483

**Published:** 2023-05-25

**Authors:** Nikita A. Sobolev, Konstantin S. Larionov, Darya S. Mryasova, Anna N. Khreptugova, Alexander B. Volikov, Andrey I. Konstantinov, Dmitry S. Volkov, Irina V. Perminova

**Affiliations:** 1 Department of Chemistry, Lomonosov Moscow State University, Leninskie Gory 1-3, 119991 Moscow, Russia; 2 Department of Chemistry and Physical Chemistry of Soils, Federal Research Centre, V.V. Dokuchaev Soil Science Institute, Pyzhevsky per., 7/2, 119017 Moscow, Russia

**Keywords:** humic substances, Yedoma ice complex, alas, Arctic, potentially toxic elements, Langmuir model, Sips model, ^13^C NMR, metals, complexation

## Abstract

Pollution of the Arctic Ocean by potentially toxic elements (PTEs) is a current environmental problem. Humic acids (HAs) play an important role in the regulation of PTE mobility in soil and water. The permafrost thaw releases ancient organic matter (OM) with a specific molecular composition into the Arctic watersheds. This could affect the mobility of PTEs in the region. In our study, we isolated HAs from two types of permafrost deposits: the Yedoma ice complex, which contains pristine buried OM, and the alas formed in the course of multiple thaw–refreezing cycles with the most altered OM. We also used peat from the non-permafrost region as the recent environmental endmember for the evolution of Arctic OM. The HAs were characterized using ^13^C NMR and elemental analysis. Adsorption experiments were conducted to assess the affinity of HAs for binding Cu^2+^ and Ni^2+^. It was found that Yedoma HAs were enriched with aliphatic and N-containing structures as compared to the much more aromatic and oxidized alas and peat HAs. The adsorption experiments have revealed that the peat and alas HAs have a higher affinity for binding both ions as compared to the Yedoma HAs. The obtained data suggest that a substantial release of the OM from the Yedoma deposits due to a rapid thaw of the permafrost might increase the mobility of PTEs and their toxicity in the Arctic Ocean because of much lesser “neutralization potential”.

## 1. Introduction

Environmental pollution by potentially toxic elements (PTEs) is nowadays an urgent global problem. PTEs are persistent environmental pollutants that do not degrade over time and are permanently present in the environment. Economic growth, together with transition to the green and renewable technologies and energy sources, has triggered a rapid increase in the consumption and production of copper, nickel, steel and other metals [1,2,3]. Emissions of copper and nickel smelter plants are significant sources of these elements to the environment [4,5,6]. This pollution is particularly critical in the sensitive Arctic ecosystems [7].

Although the sources of anthropogenic emissions are usually located outside the Arctic territories and their presence is caused by atmospheric transport from southern latitudes, significant industrial emissions from Cu and Ni smelters are observed in the Russian Arctic [8,9,10,11]. The levels of emission of inorganic elements into the atmosphere from the mining and processing plants of the Kola and Taimyr peninsulas of Russia were 350.8 and 427 (Ni) and 798 and 474 (Cu) t/year, respectively, in 2001–2010 [12]. These amounts account for approximately 128% (nickel) and 196% (copper) of the annual emissions of these elements in Central Europe. Such high levels of PTE emissions have a negative impact on the flora and fauna of the region. While the highest concentrations of PTEs are found in the vicinity of the emitting sources and accumulate in bottom sediments, peatlands and permafrost soils, their mobilization poses a potential threat to the global environment [7,13,14,15].

The mobility of metals in soil and water is regulated by hydrological parameters and soil regime (e.g., salinity, pH, temperature), redox conditions, chemical forms of the elements and organic matter (OM) [14,16,17,18,19]. Among the many factors that affect PTE mobility, the complexation of metals with organic matter and specifically with humic substances (HSs) is one of the most crucial [16,20,21]. Humic substances are complex molecular systems, which are ubiquitous in the environment. HSs are defined based on their solubility characteristics. Humic acids (HAs) are not soluble at pH below 2, whereas fulvic acids (FAs) are soluble in the entire pH range [22]. PTEs form stable insoluble complexes with HAs by interacting with carboxylic, phenolic and heteroatom-containing functional groups of HAs [23,24,25,26]. The molecular composition of HAs varies significantly and depends on their origin. Many studies showed that molecular composition significantly affects the sorption capacity and stability of HA complexes with PTEs [27,28,29,30,31].

Peat and permafrost OM constitute a significant part of Arctic organic matter. Permafrost alone constitutes more than half of the Earth’s soil organic carbon [32,33]. The molecular composition of peat unaffected by permafrost and permafrost OM can exhibit significant variations primarily due to the persistent low temperatures that restrict microbial transformations of permafrost OM [34,35,36]. However, recent climate warming triggered the mobilization of old organic carbon which was stored in permafrost for millennia. The rates of permafrost thawing are estimated on the order of 0.8–1.1 PgC per year [37]. This substantial release of organic carbon characterized by its unique molecular composition is now increasingly intruding into the Arctic watersheds, resulting in a shift towards older permafrost dominant OM [38,39]. This change in the molecular composition of OM could potentially lead to a change in PTE complexation and affect PTE presence and mobility in Arctic water and soil ecosystems. However, the knowledge regarding the complexation of permafrost humic acids with PTEs in the Russian Arctic, such as copper (Cu) and nickel (Ni), is very limited. Furthermore, the differences in the accumulation of PTEs compared to peat humic acids are not well understood.

Our study aims to fill the gap in the literature by investigating the impact of permafrost thawing on the mobility and complexation of potentially toxic elements with HAs in the Russian Arctic. We compare the complexation of Cu and Ni with humic acids derived from permafrost and peat soils. Our study focuses on a significant and novel factor: the shifting molecular composition of organic matter in Arctic watersheds resulting from permafrost thawing. This research aims to shed light on the potential impacts of permafrost thaw on the mobility and complexation of potentially toxic elements in Arctic ecosystems.

## 2. Materials and Methods

### 2.1. Sampling Site

The experiments were conducted on peat and permafrost HAs. A peat sample (PE) was collected from the ridge–lake complex of high moor Ilas sphagnum peat bog in the Arkhangelsk region of Russia (64.334170° N, 40.609598° E) from the 10–50 cm layer using a stainless-steel peat corer. Two permafrost samples (DY1 and DY2) were collected from the Duvanny Yar deposit near Cherskii settlement, the Republic of Sakha (Yakutia), Russia, using a UKB-12/25 rotary drill rig. The DY1 sample was collected from thermokarst depressions considered to be alas deposits characterized by multiple thaw–refreezing cycles (68.746207° N, 161.388074° E). The DY2 sample was collected from an organic-rich Pleistocene age ice-rich Yedoma permafrost deposit from layers not affected by thawing (68.744825° N, 161.383834° E). The samples were kindly provided by Dr. S.P. Davydov, the North-Eastern Research Center, the Far East Branch of RAS. Approximately 5 kg of each sample was collected and placed in a zip-lock plastic bag. The samples were frozen after collection and transported to the laboratory while still frozen. They were then stored at −25 °C in a freezer until lyophilization. In total, three samples, namely one peat sample (PE) and two permafrost samples (DY1 and DY2), were used in this study.

The characteristics of peat and permafrost samples such as pH, organic carbon (OC) and total nitrogen (TN) concentrations and initial moisture content (MC) are presented in Table 1.

### 2.2. Extraction of Humic Acids

The extraction of HAs was carried out from lyophilized peat and permafrost samples. Extraction of nonpolar organic components was conducted using a 1:1 *v*/*v* (hexane:ethanol) mixture in a Soxhlet extractor for 4 h. After that, the samples were dried in a vacuum oven at 40 °C for 12 h. Then, decalcification was carried out with 1 M HCl (1:10 solid:liquid solution phase weight ratio) until the pH value dropped down to 2. The acidified suspension was shaken for 6 h and left for 24 h [40]. The residual decalcified samples were neutralized by adding 1 M NaOH to pH 7, and then 0.1 M NaOH was added (1:10 sample:solution weight ratio) according to the International Humic Substances Society (IHSS) recommendations [41]. Then, NaCl was added until the final concentration of 0.3 M NaCl to the combined extract, which was centrifuged for 20 min at 10,000 rpm to coagulate colloidal particles. The supernatant was then separated from the precipitate and acidified to pH 1–2 with 1 M HCl. The samples were then left to precipitate for 24 h and centrifuged at 10,000 rpm. Purification of precipitated HA gels from silicates and ash components was carried out with a mixture of 0.1 M HCl and 0.3 M HF for 12 h. Then, the HA samples were purified by dialysis (3.5 kDa membrane) until a negative test for Cl^−^ with AgNO_3_. The purified HA samples were freeze-dried, homogenized with mortar and pestle, and sieved through a sieve with d = 0.1 mm. All extraction and purification procedures, excluding dialysis, were performed under argon to prevent oxidation.

### 2.3. Characterization of the Initial Samples and Isolated Humic Acids

Quantitative ^13^C solution state NMR spectra were acquired using an Avance-400 BioSpin spectrometer (Bruker, Bremen, Germany) operating at 400 MHz proton frequency. For the NMR study, 40 mg of HA was dissolved in 1 mL of NaOD in 40 wt. % in D_2_O, 99.5 atom % D (Sigma Aldrich, Germany), and ^13^C NMR spectra were recorded according to the conditions described in [42,43]. To exclude the nuclear Overhauser effect, the INVGATE pulse technique was used. For complete carbon nuclei relaxation, the pulse delay was set to 8 s, and the number of scans averaged was 4500. All NMR spectra were acquired after centrifugation using a 5 mm broadband probe.

Moisture content was determined using an MX-50 Moisture Analyzer (AND, Tokyo, Japan). Soil pH was analyzed using the 9045D EPA method [44] using a SevenCompact pH/Ion meter and glass electrode InLab Expert Pro (Mettler Toledo, Columbus, OH, USA). OC and TN concentrations as well as mass concentrations of CHNS and O were analyzed using a 2400 Series II CHNS/O elemental analyzer (PerkinElmer, Waltham, MA, USA) according to [45].

### 2.4. Adsorption Experiments

The sorption kinetic experiments were carried out at 25 ± 0.5 °C, pH 5 ± 0.03 and 0.01 M KNO_3_. The weights of 15,000 ± 0.010 mg HA were measured using a BM-22 analytical balance (AND, Tokyo, Japan) and placed into 15 mL centrifuge tubes (Sarstedt, Nümbrecht, Germany). Aliquots of 10 mL of solution containing 20 mg/L Cu^2+^ or 15 mg/L Ni^2+^ were added into the tubes, which were placed onto an overhead shaker at 75 rpm (multi-rotator RS-60 (Biosan, Riga, Latvia)). The sorption was measured at different time intervals (5, 10, 15, 25, 30, 45, 60 and 90 min). Adsorption isotherm experiments were carried out at 25 ± 1 °C, pH 5 ± 0.03 and 0.01 M KNO_3_. Then, 15 mg of HA and 10 mL solution containing 1 –100 mg/L Cu^2+^ or 0.5–100 mg/L Ni^2+^ were shaken for 8 h at 25 ± 1 °C at 75 rpm. The effect of pH was assessed by adjusting the solution pH within the range of 2–6.5 using the same conditions as the isotherm experiments. pH adjustment was conducted using 0.1 M solutions of KOH and HNO_3_ which were added dropwise to each solution.

Measurements were conducted using an axial ICP-AES 720-ES spectrometer (Agilent Technologies, Santa Clara, CA, USA) equipped with a low-flow axial quartz torch. The instrument was calibrated within the range of 0.025–10 mg/L. It was equipped with a 2.4 mm inner diameter injector tube (Glass Expansion, Melbourne, Australia), a double-pass glass cyclonic spray chamber (Agilent Technologies) and a OneNeb nebulizer (Agilent Technologies, Santa Clara, CA, USA). A Sc solution (20 ppm) prepared from 1000 mg/L Sc standard solution (High Purity Standards, North Charleston, SC, USA) was introduced online together with a sample solution using Trident Internal Standard Kit (Glass Expansion) and used as an internal standard. Emission line 361.383 nm was used for scandium as an internal standard correction. A 1 ppm QC standard solution was prepared from QCS-27-250 (High Purity Standards) and used to control accuracy and calibration stability after each batch of adsorption experiments. A 5% HNO_3_ solution was used to control the blank signal to assess possible memory effects. The measurement results obtained on different lines were averaged with differences between them of less than 5% and were used for final calculations. All glass and polypropylene vessels were thoroughly rinsed using concentrated nitric acid and deionized water before adsorption and analysis procedures. Levels of all test elements in the blank samples were below their limits of detection (LODs).

Conditions of ICP–AES measurements are shown in Table 2. All lines were measured simultaneously (a MultiCal mode). Linear and rational (quadratic) functions were used for the calibration. The position and baseline corrections were performed for all the peaks of distinctive elements using the tools of the spectrometer software.

Deionized water (18.2 MΩ × cm from a Milli-Q Academic system, Millipore, Molsheim, France) was used to prepare all the solutions and for washing.

All initial concentration solutions were treated the same way as samples to assess the possible sorption on vial walls and errors during initial dilution. The obtained concentration values were used as initial concentrations (C_0_).

The pH of the solutions was analyzed using SevenCompact pH/Ion meter and glass electrode InLab Expert Pro (Mettler Toledo, Columbus, OH, USA) calibrated using 1.68, 4.01, 6.85 and 9.18 buffer calibration solutions (Hanna Instruments, Woonsocket, RI, USA).

The amount (mg) of Me^2+^ ions adsorbed at equilibrium per gram of HA was calculated using the following equation:

(1) $$q_{e}=(C_{0}-C_{e})\;\cdot V/m,$$

where q_e_ is the quantity of Me^2+^ ions adsorbed at equilibrium (mg/g), C_0_ is the initial concentration of Me^2+^ ions (mg/L), C_e_ is the equilibrium concentration of Me^2+^ ions (mg/L), V is the volume of the metal solution (L) and m is the weight of HA (g).

The adsorption degree was calculated according to Equation (2):

(2) $$\eta =\frac{\left(C_{0}-C_{e}\right)}{C_{0}}\cdot 100,$$

where η is the adsorption degree (%).

The kinetics of Cu^2+^ and Ni^2+^ sorption was examined under batch conditions for a period of 90 min, and the acquired kinetic data were analyzed using kinetic models, namely the Lagergren pseudo-first-order (PFO) kinetic model [46], the Ho and McKay, or pseudo-second-order (PSO), kinetic model [47,48], and the Weber–Morris (W–M) intraparticle diffusion kinetic model [49], as shown in Equations (3)–(5), respectively.

(3) $$q_{t}=q_{e}(1-e^{\left(-k_{1}\cdot t\right)}),$$

(4) $$q_{t}=\frac{k_{2}\cdot {q_{e}}^{2}\cdot t}{1+k_{2}\cdot q_{e}\cdot t},$$

(5) $$q_{t}=k_{id}\cdot t^{1/2}+C_{id}$$

where t is the time; q_e_ and q_t_ are the amounts of adsorbed metal ions per mass of sorbent (mg/g) at equilibrium and at time t, respectively; k_1_ is the pseudo-first-order rate constant (min^−1^); k_2_ is the pseudo-second-order rate constant (g·mg^−1^⋅min^−1^); k_id_ is the intraparticle diffusion rate constant (g·mg^−1^·min^−1/2^); and C_id_ is the parameter reflecting an effect of the boundary layer thickness (mg·g^−1^).

The adsorption isotherms were constructed by fitting the experimental data to non-linear Langmuir [50], Freundlich [51], Dubinin–Radushkevich (D-R) (equation adapted for adsorption from aqueous solution) [52] and Sips [53] adsorption models for both Cu^2+^ and Ni^2+^. To fit these models and to calculate the parameters of the adsorption, the corresponding Equations (6)–(9) were used:

(6) $$q_{e}=\frac{q_{m}\cdot K_{L}\cdot C_{e}}{1+K_{L}\cdot C_{e}},$$

(7) $$q_{e}=K_{F}\cdot {C_{e}}^{1/n},$$

(8) $$q_{e}=q_{m}\cdot exp(-\beta \cdot {\left(R\cdot T\cdot \ln\left(C_{s}/C_{e}\right)\right)}^{2}),$$

(9) $$q_{e}=\frac{q_{m}\cdot K_{s}\cdot {C_{e}}^{n}}{1+K_{s}\cdot {C_{e}}^{n}},$$

where q_e_ is the equilibrium and q_m_ is the maximum adsorption capacity of metal ions (mg/g); C_e_ is the equilibrium concentration of metal ions (mg/L); K_L_ is the Langmuir constant (L/g); K_F_ is the Freundlich constant (L/g); n is the indicator of the surface heterogeneity degree; β is activity coefficient which represents the adsorption energy (mol^2^·kJ^−2^), from which mean free energy was calculated as E = $\sqrt{1/\beta^{2}}$ (kJ/mol); C_s_ is the solubility (mg/L); R is the molar gas constant (J/(mol⋅K)); T is the temperature (K); K_S_ is the Sips constant related to the energy of adsorption (L/g); and n is the Sips model exponent, which describes the heterogeneity of the adsorption sites on the sorbent surface.

Adsorption kinetic curves and isotherms were plotted using the Python library matplotlib and SciPy. The same open-source software was used to determine sorption parameters [54]. To investigate the effect of different error functions on the isotherms measured, six error functions, namely r^2^, HYBRID, mean percent standard deviation (MPSD), average relative error (ARE), sum of squared errors (SSE) and average absolute error (EABS), were evaluated. The respective methods are described elsewhere [55]. The isotherm parameters were obtained by minimizing the error functions within the range of the studied concentrations. The analysis was conducted using the Python libraries and code mentioned above.

All experiments were conducted in triplicates, and the average values are reported.

## 3. Results and Discussion

### 3.1. Characterization of the Samples of Humic Acids Isolated in this Study

The results of structural group analysis (^13^C NMR) and elemental composition of the three isolated HA samples are summarized in Table 3.

According to the ^13^C NMR data obtained, the PE and permafrost samples differed substantially in the content of oxygen-containing carboxylic, carbonylic and phenolic groups (we designated them as ΣOx) which are mostly responsible for metal ion complexation [56,57]. The HAs isolated from alas deposits (DY1) has the highest ΣOx value of 26.7% C, which is 1.5% higher than PE (25.2% C) and more than 5% higher as compared to DY2 (21.6% C).

However, the contents of carbohydrate fragments in the region of 55–112 ppm for both alas and Yedoma permafrost HAs DY1 and DY2 are 36% and 35%, respectively, which are higher than that for PE HA (33%). This difference in carbohydrate content can be attributed to the process of biodegradation, which is a primary pathway for carbohydrate decomposition. The peat sampling area is located in a warmer climate than permafrost. Higher temperatures, absence of permanently frozen layers, greater variety and abundance of microbial community lead to a higher rate of carbohydrate decomposition [58] and, as a result, a lower amount of carbohydrates in PE HAs.

The lowest overall distribution of C among oxygen-containing functional groups (Σ of 47–112 and 146–220 ppm region) was found for DY2 HA (63%), while PE HA (65%) and DY1 (68%) HAs had a relatively higher portion of C bonded to oxygen. This is supported by the elemental analysis data, specifically the O/C ratio which is higher for DY1 and PE (0.48 and 0.46) as compared to DY2 (0.41).

The aromaticity of HA C_ar_/C_Alk_ was calculated as a ratio of spectral intensity of C atoms of aliphatic structures within a range from 0 to 112 ppm to the sum of aromatic structure C signals within a range from 112 to 166 ppm [59]. DY1 and PE were characterized by the higher values of C_Ar_/C_Alk_ of 0.61 and 0.57, respectively, as compared to the sample of DY2 HA (0.50). This could be explained by the deeper oxidation and biodegradation of easily degradable aliphatic fragments in this permafrost sample [58,60,61]. The H/C ratio, which is also an indicator of aliphaticity [59], is approximately 10% higher for DY2 than for DY1 and PE HA (1.18, 1.09 and 1.07 respectively), which confirms their more aromatic structure.

The C/N ratio of 13.9 is significantly lower for DY2 HA, while PE and DY1 have C/N ratios of 17.1 and 17.2, respectively. The lower values of the C/N ratio and the higher content of nitrogen in the Yedoma permafrost HA sample may be connected with lower rates of microbial nitrogen consumption [62] or the changes in the vegetation during the formation of permafrost layers, as *sphagnum* spp. which are presently common for bogs have a higher C/N ratio than flora which was common in fens [63].

The concentration of another heteroatom in HA molecular composition, sulfur (S), differs insignificantly between all samples, with an S/C ratio of 0.011 ± 0.002.

We found that the alas and peat HA samples have the highest values of aromaticity and oxygen-containing functional groups. This indicates a higher proportion of strong metal-binding functional groups in the molecular composition of these two samples. The Yedoma sample is characterized by the more reduced character. This may be related to its significantly lower exposure to oxidation and biodegradation, as the Yedoma (DY2) permafrost deposit was below the active layer border and was not affected by thawing, oxidation and enhanced biodegradation in comparison with alas (DY1) and peat (PE) humic acids.

### 3.2. Effect of pH on Cu^2+^ and Ni^2+^ Adsorption on Humic Acids

**Figure 1:**
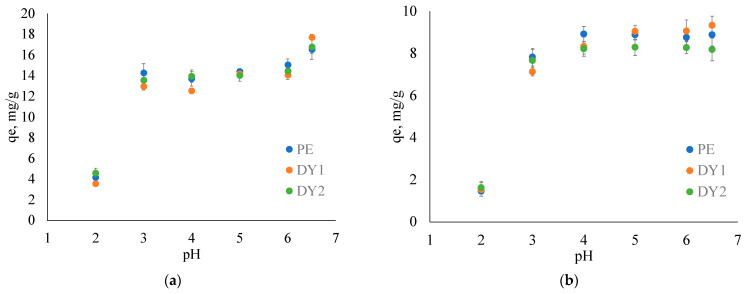
Effect of pH on equilibrium adsorption capacity of (**a**) Cu^2+^ and (**b**) Ni^2+^ ions on PE, DY1 and DY2 HAs (t = 25 °C).

The negative impact on the environment from Cu and Ni smelters not only is connected to the release of toxic levels of PTEs, but also brings about the high emissions of SO_2_ gas, which may cause a significant decrease in the pH of the affected water and soil ecosystems [64,65,66,67]. A decrease in pH may affect the mobility of the elements in the environment due to its leaching from inorganic and organic complexes [68]. The typical pH values of the Arctic rivers and lakes vary in a wide range from 5 to 9 [69], while in Arctic regions affected by Ni and Cu smelter emissions, typical pH values drop down to 5–7 [7], and sometimes to 4 [70]. This is why we studied the effect of pH on Cu^2+^ and Ni^2+^ adsorption degree on isolated HAs in the pH range of 2 to 6.5. Figure 1 shows the dependence of adsorbed ions of elements at equilibrium on the pH of the solution.

From Figure 1, it can be observed that the equilibrium concentration of both elements decreased as the pH decreased for all three humic acid (HA) samples. The Cu^2+^ adsorption degree decreased from its maximum value of (16 ± 1) mg/g at pH 6.5 down to 12–14 mg/g within a pH range from 3 to 6. An abrupt decrease was observed at pH 2: the corresponding q_e_ accounted for 3.5–4.5 mg/g. On the contrary, Ni^2+^ adsorption did not depend significantly on pH in the range of 4 to 6.5 (the q_e_ value varied from 8.1 up to 9.3 mg/g for all HA samples). Still, at pH < 4, there was an abrupt reduction observed down to 1.4–1.6 mg/g.

The increase in Cu adsorption degree at pH 6.5 (Figure 1a) can be attributed to the transformation of Cu species in solution at pH > 5 from Cu^2+^ to CuOH^+^ and Cu(OH)_2_ and its precipitation at pH 6.5 where Cu(OH)_2_ formation is somewhat more pronounced [71,72]_._ In contrast, Ni was presented as Ni^2+^ in the whole studied pH range [73], and its adsorption was not significantly affected in the pH range from 4 to 6.5 (Figure 1b). The decrease in the adsorption degree of HAs at pH < 3 for both ions may be associated with the competition between the protonation of carboxylic groups of HAs which gradually dissociates at pH 2.5–7 and the adsorption of metal ions [31]. The results showed that the difference in the pH between 4 and 6 which is typical for acidified lakes and rivers near Cu and Ni smelters does not significantly affect the adsorption degree of these ions. Therefore, a pH 5 was selected for further adsorption studies.

### 3.3. Adsorption Kinetics and Isotherms

To assess the rate of Cu^2+^ and Ni^2+^ adsorption on humic acids, kinetic experiments were conducted. Figures S1 and S2 show the relationships between the amount of Cu^2+^ and Ni^2+^ adsorbed on HA and time. The experimental results were fitted using the PFO, PSO and W-M adsorption kinetic models. The calculated model parameters are outlined in Table 4.

The adsorption process of Cu^2+^ and Ni^2+^ proceeds at relatively high rates. Data show a Cu-rich equilibrium for 30–45 min. The calculated q_e_ was 14.4, 15.4 and 14.1 for PE, DY1 and DY2, respectively. The results for model fitting showed that the pseudo-second-order model better describes the kinetics of Cu^2+^ adsorption onto all three HA sorbents. This indicates that the limiting stage of Cu ion adsorption onto HAs is chemosorption. The adsorption rate of Ni^2+^ was found to be even faster. The equilibrium concentration q_e_ = 8.6 and 9.2 mg/g was achieved faster for PE and DY1 HAs, respectively, already after 10 min of adsorption reaction. For DY2, the equilibrium was achieved at 20 min with q_e_ = 7.8 mg/g. These high rates of Ni adsorption negatively influenced the results of the kinetic modeling as there were limited points for curve fitting (Figure S2). However, the fitting of the models showed that the PFO kinetic model describes Ni adsorption in each HA with r^2^ 0.89 for all HAs. The PFO model assumes that the initial adsorption rate is fast and that the rate of adsorption decreases as the amount of adsorbate on the HA surface approaches equilibrium. The rapid adsorption rates observed in our study are consistent with the findings of Lodygin et al., who reported that the adsorption equilibrium of Cd^2+^ and Zn^2+^ ions on permafrost humic acids is reached within 30 min [74]. On the basis of the obtained kinetic results, it was also concluded that 8 h is sufficient for the complete establishment of sorption equilibrium for both ions.

An adsorption isotherm study was conducted to determine the maximum adsorption capacity (q_m_) of HSs for a particular metal ion and the affinity of HSs for that metal ion.

**Figure 2:**
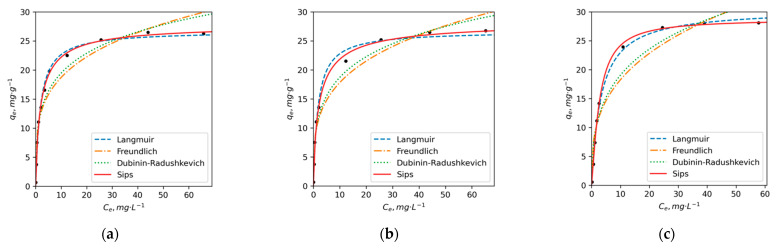
The adsorption isotherms of Cu^2+^ ions on (**a**) PE, (**b**) DY1 and (**c**) DY2 HAs in non-linear coordinates of Langmuir, Freundlich, Dubinin–Raduschkevich and Sips adsorption equations (pH = 5, t = 25 °C).

The calculated parameters for each model are presented in Table 5. It can be noted that for both ions and each HA sample, the determination coefficient r^2^ was ≥0.90. This indicates that each model could be used to explain the adsorption behaviors. To properly evaluate the accuracy of a model in fitting experimental data, HYBRID, MPSD, ARE, SSE and EABS error functions together with r^2^ were used. According to the obtained results, the Sips and Langmuir models demonstrated higher r^2^ and lower error functions compared to the other models. The results of error function calculations are presented in Table S1. Based on these findings, Sips and Langmuir models were selected as suitable models for describing the adsorption process of Cu^2+^ on HAs. Figure 2 illustrates the adsorption isotherms for Cu^2+^ and three HAs, confirming that both models provide a good fit to the experimental data.

In the case of the Langmuir adsorption model, it can be stated that the surface of HAs contains a finite number of identical sites available for adsorption, and the adsorption of Cu^2+^ ions does not affect one another. According to the Langmuir model, Cu^2+^ ions form a monolayer coverage on the surface of HAs without any interaction between the adsorbed ions [75,76]. At the same time, the Sips isotherm model, which combines the Langmuir and Freundlich models, is known to provide a better description of heterogeneous surfaces. Specifically, at low sorbate concentrations, it reduces to a Freundlich isotherm, while at high sorbate concentrations, it predicts a monolayer adsorption capacity consistent with the Langmuir isotherm [77,78].

The calculated q_m_ parameters for Cu adsorption using both models show minimal differences, with Sips q_m_ fluctuating around 28 ± 1 mg/g. However, the Langmuir model yields a higher q_m_ value for DY2 (31 mg/g), whereas the q_m_ values for PE and DY1 are approximately 27 mg/g. The K_L_ and K_S_ values for PE and DY1 HAs are also quite similar, with 0.56 and 0.55 for PE and 0.58 and 0.52 (L/g) for DY1, respectively. On the other hand, DY2 exhibits significantly lower K_L_ and K_S_ values, at 0.30 and 0.29 L/g, respectively. The higher q_m_ and lower K_L_ and K_S_ values observed for DY2 suggest that while it has a slightly higher capacity for Cu^2+^ adsorption, it also displays a lower affinity for Cu ions. This implies that DY2 may form less stable complexes with Cu^2+^ compared to DY1 and PE.

It is worth noting that the adsorption degree of Cu ions by all humic acids (HAs) in the ratios of dissolved organic carbon (DOC) to Cu ~100–1000:1 was observed to be 94 ± 1% for PE, 92 ± 2% for DY1 and 85 ± 5% for DY2. These ratios are specific to Arctic lakes and rivers that have been exposed to Cu pollution. In these environments, the concentration of DOC typically ranges from 1 to 10 mg/L or approximately 2 to 20 mg/L of HAs, while the concentration of Cu is around 1–20 µg/L [7,79,80].

**Figure 3:**
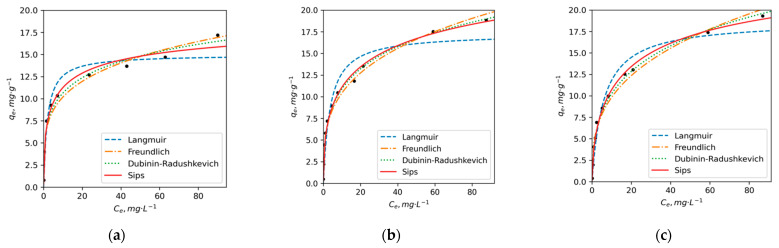
The adsorption isotherms of Ni^2+^ ions on (**a**) PE, (**b**) DY1 and (**c**) DY2 HAs in non-linear coordinates of Langmuir, Freundlich, Dubinin–Raduschkevich and Sips adsorption equations (pH = 5, t = 25 °C).

The aforementioned observations suggest that DY2 exhibits a slightly lower adsorption capacity for Cu compared to PE and DY1. These findings align with the results of elemental and NMR analyses, which indicated higher concentrations of oxygen, higher O/C ratios, greater aromaticity and a higher proportion of C atoms bonded to oxygen for PE and DY1. In contrast, DY2 is more aliphatic and possesses a lower content of oxygen-containing groups that are primarily responsible for metal adsorption and chelation. The adsorption isotherms of Ni^2+^ ions exhibit notable differences from the Cu^2+^ curves (Figure 3). The error analysis results (Table S2) indicate that the Sips and D-R models provide better predictions for the adsorption of Ni compared to the Langmuir and Freundlich models. These two models demonstrate higher r^2^ values and are characterized by lower error function values. A good fit of the D-R model for Ni ions suggests a complex mechanism of adsorption that involves both chemosorption and physical sorption in the pores of HAs.

According to the results presented in Table 5, the maximum adsorption capacities obtained for Ni ions by D-R model follow the order PE (31) < DY1 (44) < DY2 (52) (mg/g). The calculated values of q_e_ using Sips model are lower: 20, 32 and 27 mg/g for PE, DY1 and DY2, respectively. The K_S_ of Ni^2+^ adsorption on PE HA is more than 2 times higher (0.41) than that for DY2 (0.19) and DY1 (0.17 L/g). This indicates that Ni has a higher affinity to PE than to the permafrost HAs. The mean free energy obtained by the calculation of D-R model parameters for both elements lies within 13–16 kJ/mol, which indicates that the adsorption of Ni and Cu on HAs follows a chemical ion-exchange mechanism [81].

The average values of Ni concentration in Arctic waterbodies affected by Ni pollution are generally lower compared to Cu in freshwater reservoirs of the Kola and Taymyr peninsulas; the average concentrations of Ni range from 0.9 to 4.3 µg/L, with levels reaching up to 147 µg/L near emission sources [7,80]. These concentrations correspond to ratios of approximately 100–1000:1 (OM:Ni). Under these conditions, the adsorption degree of HAs to Ni is 91 ± 5%, 89 ± 4% and 82 ± 3% for PE, DY1 and DY2, respectively, while at the ratio ~ 10:1 at extremely low values of DOM concentration in water and/or high Ni pollution, the adsorption degree decreases to 21 ± 1% for all HA samples.

Overall, higher and more consistent q_m_ values, as well as sorption constants, were observed for Cu compared to Ni when adsorbed onto the three HA samples using all four models employed in this study. Higher K_S_, K_L_ and K_F_ values were obtained for the PE HA sample, followed by DY1 and then by DY2. The latter sample has significantly lower metal binding affinity as compared to more oxidized OM.

## 4. Conclusions

We found that the HA samples isolated from the permafrost deposit had very different properties: the alas HA sample, which we defined as “the oxidized PM HA” (DY1), had properties very similar to the HA sample from peat, whereas Yedoma permafrost HA (DY2) was characterized by a much more reduced nature due to the higher contribution of the saturated aliphatic structures.

The adsorption experiments have revealed that the more oxidized and aromatic HA samples from the non-permafrost peat and from the alas permafrost had a higher affinity for binding both copper and nickel ions as compared to the well-preserved Yedoma permafrost HAs. The obtained data suggest that a substantial release of the organic matter from the Yedoma deposits due to a rapid thaw of the permafrost might bring about an increase in the mobility of PTEs and in their toxicity in the Arctic Ocean because of much lesser “neutralization potential”. This factor should be taken into consideration for risk assessment scenarios while predicting the consequences of climate change on the pollution of the Arctic region.

## Figures and Tables

**Table 1:** The characteristics of the peat and permafrost samples, including pH (t = 25 °C), OC and TN concentrations in g/kg of absolute dry sample and initial moisture content in %.

Sample	pH	OC (g/kg)	TN (g/kg)	MC (%)
PE	3.52 ± 0.08	478 ± 2	12.2 ± 0.4	84 ± 7
DY1	7.54 ± 0.10	22.1 ± 0.7	2.4 ± 0.1	28 ± 2
DY2	7.60 ± 0.06	11.1 ± 0.3	1.3 ± 0.1	22 ± 3

**Table 2:** Conditions of ICP–AES measurements.

Conditions for All Line Registrations	
RF power (kW)	1.40
Plasma flow (L/min)	18.0
Axial flow (L/min)	1.50
Nebulizer flow (L/min)	1.00
Replicate read time (s)	20
Instrument stabilization delay (s)	15
Replicates	4
Sample uptake delay (s)	25
Pump rate (rpm)	12

**Table 3:** The carbon distribution among the structural fragments of HA obtained by ^13^C NMR signal integration (% of total carbon); calculated ΣOx and C_ar_/C_alk_ coefficients and elemental ratios obtained from the results of elemental analysis.

Sample	CHn	CH_3_O	CH_n_O	OCO	C_ar_	C_ar_O	COO	C=O	ΣOx	C_ar_/C_alk_	H/C	O/C	C/N
PE	18.0	7.1	23.1	9.9	23.8	9.1	11.5	4.6	25.2	18.0	1.07 ± 0.01	0.46 ± 0.01	17.2 ± 0.1
DY1	12.9	5.3	27.2	8.9	24.3	9.0	12.4	5.3	26.7	12.9	1.09 ± 0.02	0.48 ± 0.01	17.1 ± 0.1
DY2	19.1	6.6	26.9	8.3	24.1	6.6	11.2	3.8	21.6	19.1	1.18 ± 0.01	0.41 ± 0.01	13.9 ± 0.1

**Table 4:** Kinetic parameters and correlation coefficients of Cu^2+^ and Ni^2+^ ion adsorption onto PE, DY1 and DY2 HAs obtained by fitting of PFO, PSO and Webber–Morris non-linear kinetic models.

		Cu			Ni		
Sample		PE	DY2	DY1	PE	DY2	DY1
PFO	k_1_	0.35	0.38	0.37	0.45	0.47	0.52
	r^2^	0.72	0.62	0.59	0.99	0.91	0.89
PSO	k_2_	0.089	0.083	0.14	0.32	0.29	0.47
	r^2^	0.90	0.90	0.92	0.63	0.70	0.69
W-M	C_id_	12	13	8.8	7.8	8.8	7.5
	k_id_	0.25	0.25	0.16	0.067	0.075	0.049
	r^2^	0.64	0.61	0.69	0.28	0.34	0.36

**Table 5:** The Langmuir, Freundlich, Dubinin–Radushkevich and Sips isotherm parameters and correlation coefficients (r^2^) for adsorption of Cu^2+^ and Ni^2+^ ions onto PE, DY1 and DY2 HAs (pH = 5).

Sample		Cu			Ni		
		PE	DY2	DY1	PE	DY2	DY1
Langmuir	q_m_, mg/g	27	27	30	15	17	19
	K_L_, L/g	0.56	0.58	0.30	0.52	0.28	0.17
	r^2^	0.99	0.99	0.99	0.94	0.93	0.96
Freundlich	n	3.84	3.67	3.10	4.3	3.5	3.0
	K_F_, L/g	10	9.5	8.7	6.0	5.3	4.6
	r^2^	0.90	0.94	0.90	0.96	0.98	0.97
D-R	q_m_, mg/g	64	65	88	31	44	52
	E (KJ/mol)	15	15	14	16	14	13
	r^2^	0.94	0.96	0.93	0.97	0.99	0.99
Sips	q_m_, mg/g	28	29	29	20	32	27
	K_S_, L/g	0.55	0.52	0.29	0.41	0.19	0.17
	n	0.85	0.80	1.26	0.49	0.45	0.58
	r^2^	0.99	0.99	0.99	0.98	0.99	0.99

## Data Availability

Data are available upon request to the corresponding author.

## Supplementary Materials

The following supporting information can be downloaded at https://www.mdpi.com/article/10.3390/toxics11060483/s1: Figure S1: Kinetic curves of Cu^2+^ adsorption on HAs; Figure S2: Kinetic curves of Ni^2+^ adsorption on HAs; Table S1: The results of error function calculations for Cu^2+^; Table S2: The results of error function calculations for Ni^2+^.
