# Supplementary material for: Yedoma Permafrost Releases Organic Matter with Lesser Affinity for Cu2+ and Ni2+ as Compared to Peat from the Non-Permafrost Area: Risk of Rising Toxicity of Potentially Toxic Elements in the Arctic Ocean
Source: Toxics. 2023 May 25;11(6):483. doi: 10.3390/toxics11060483 (PMC10302542; doi:10.3390/toxics11060483)
Supplement: Supplementary file 1 [file toxics-11-00483-s001.zip › toxics-2375618-supplementary.pdf]

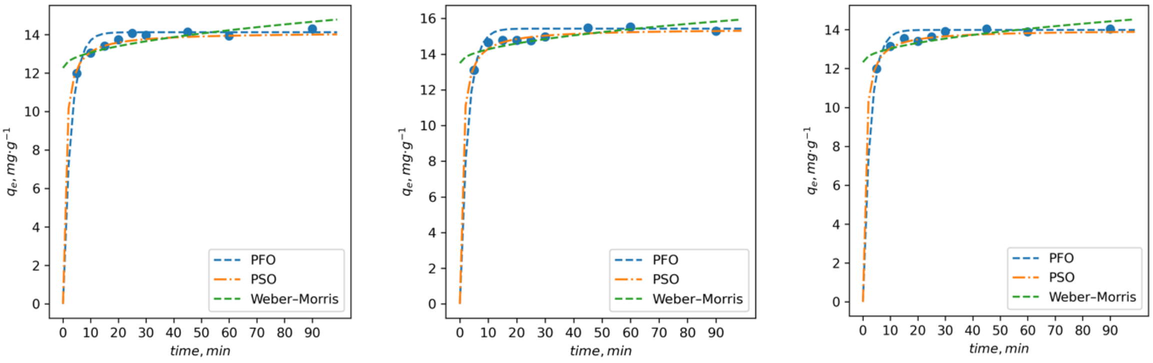

(a) **(b)** **(c)**

Figure S1. Kinetic curves of Cu^{2+} adsorption on HAs (a) PE; (b) DY1 (c) DY2.

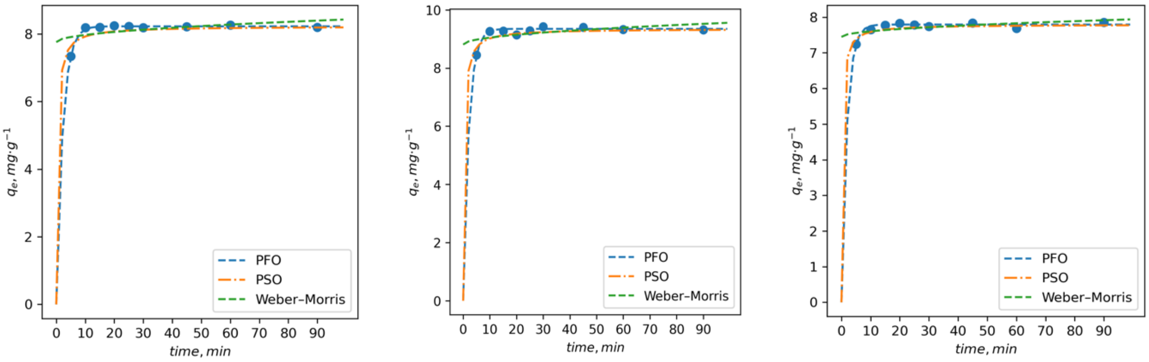

(a) **(b)** **(c)**

Figure S2. Kinetic curves of Ni^{2+} adsorption on HAs (a) PE; (b) DY1 (c) DY2.

Table S1. The results of error function calculations for Cu^{2+}.

Model	Hybrid	MPSD	ARE	SSE	EABS	r^2
PE						
Langmuir	12	31	1.16	6.9	7.4	0.99
Freundlich	503	112	8.0	87	26	0.90
D-R	313	90	6.3	55	21	0.94
SIPS	26	26	1.8	4.1	6.2	0.99
DY1						
Langmuir	16	43	1.7	8.5	7.4	0.99
Freundlich	438	107	9.7	53	19	0.94
D-R	272	83	7.7	32	15	0.96
SIPS	48	36	3.0	5.1	5.8	0.99
DY2						
Langmuir	50	34	3.5	5.3	5.9	0.99
Freundlich	654	147	13	97	26	0.90
D-R	438	125	10	70	22	0.93
SIPS	9.8	17	1.4	1.1	2.7	0.99

Table S2. The results of error function calculations for Ni^{2+}.

Model	Hybrid	MPSD	ARE	SSE	EABS	r^2
PE						
Langmuir	25	59	1.9	14	9.6	0.94
Freundlich	101	49	4.3	9.8	8.0	0.96
D-R	51	38	3.1	5.9	6.4	0.97
SIPS	27	37	2.0	4.8	5.5	0.98
DY1						
Langmuir	39	78	2.2	26	15	0.93
Freundlich	85	40	3.9	6.9	7.1	0.98
D-R	32	26	2.4	2.9	4.6	0.99
SIPS	32	29	2.3	3.2	4.8	0.99
DY2						
Langmuir	21	61	3.0	15	11	0.96
Freundlich	117	50	5.1	9.8	8.9	0.97
D-R	52	33	3.3	4.3	5.2	0.99
SIPS	31	29	2.4	2.9	4.4	0.99
